# Alterations of cerebral microcirculation in peritumoral edema: feasibility of in vivo sidestream dark-field imaging in intracranial meningiomas

**DOI:** 10.1093/noajnl/vdaa108

**Published:** 2020-08-27

**Authors:** Moncef Berhouma, Thiebaud Picart, Chloe Dumot, Isabelle Pelissou-Guyotat, David Meyronet, François Ducray, Jerome Honnorat, Omer Eker, Jacques Guyotat, Anne-Claire Lukaszewicz, François Cotton

**Affiliations:** 1 Department of Neurosurgical Oncology and Vascular Neurosurgery, Pierre Wertheimer Neurological and Neurosurgical Hospital, Hospices Civils de Lyon, Lyon, France; 2 Creatis Lab, CNRS UMR 5220, INSERM U1206, Lyon 1 University, INSA Lyon, Lyon, France; 3 Department of Pathology, Pierre Wertheimer Neurological and Neurosurgical Hospital, Hospices Civils de Lyon, Lyon, France; 4 Centre de Recherche en Cancérologie de Lyon INSERM U1052 CNRS 5286, Lyon 1 University, Lyon, France; 5 Department of Neurooncology, Pierre Wertheimer Neurological and Neurosurgical Hospital, Hospices Civils de Lyon, Lyon, France; 6 Department of Neuroradiology, Pierre Wertheimer Neurological and Neurosurgical Hospital, Hospices Civils de Lyon, Lyon, France; 7 Department of Neuroanesthesia and Neurocritical Care, Pierre Wertheimer Neurological and Neurosurgical Hospital, Hospices Civils de Lyon, Lyon, France; 8 Department of Neuroimaging, Centre Hospitalier Lyon Sud, Hospices Civils de Lyon, Pierre-Bénite, France

**Keywords:** ischemia, meningioma, microcirculation, peritumoral edema, sidestream dark-field imaging

## Abstract

**Background:**

Intracranial meningiomas display a variable amount of peritumoral brain edema (PTBE), which can significantly impact perioperative morbidity. The role of microcirculatory disturbances in the pathogenesis of PTBE is still debated. The aim of this study was to microscopically demonstrate and intraoperatively quantify, for the first time, the alterations to microcirculation in PTBE using sidestream dark-field (SDF) imaging.

**Methods:**

Adult patients with WHO grade I meningiomas were recruited over a 9-month period and divided into 2 groups depending on the absence (NE group) or the presence (E group) of PTBE. In vivo intraoperative microcirculation imaging was performed in the peritumoral area before and after microsurgical resection.

**Results:**

Six patients were included in the NE group and 6 in the E group. At the baseline in the NE group, there was a minor decrease in microcirculatory parameters compared to normal reference values, which was probably due to the mass effect. In contrast, microcirculatory parameters in the E group were significantly altered, affecting both vessel density and blood flow values, with a drop of approximately 50% of normal values. Surgical resection resulted in a quasi-normalization of microcirculation parameters in the NE group, whereas in the E group, even if all parameters statistically significantly improved, post-resection values remained considerably inferior to those of the normal reference pattern.

**Conclusion:**

Our study confirmed significant alterations of microcirculatory parameters in PTBE in meningiomas. Further in vivo SDF imaging studies may explore the possible correlation between the severity of these microcirculatory alterations and the postoperative neurological outcome.

**Key Points**

- In vivo analysis of peritumoral microcirculation is possible with sidestream dark-field imaging.
- Microcirculation parameters are significantly altered in the peritumoral edema in meningiomas.

**Importance of the Study**

Intracranial meningiomas regularly display peritumoral edema. The responsibility of microcirculation disturbances and perfusion anomalies in the pathogenesis of this peritumoral edema is still debated. Using sidestream dark-field imaging, we demonstrated and quantified for the first time in vivo the existence of significant alterations of microcirculation parameters including blood flow and vessel density in the peritumoral edema in meningiomas. We confirmed the persistence of significant microcirculation alterations after surgical resection and mass effect withdrawal. Our results support the concept of vascular vulnerability of the peritumoral brain edema. Future studies using sidestream dark-field imaging may help to establish in vivo a correlation between the intraoperative severity of peritumoral microcirculation alterations and the neurological outcome.

Meningiomas represent the second most common intracranial tumor in adults, accounting for approximately 20% of all intracranial tumors.^1^ Approximately 38–67% of meningiomas exhibit a variable amount of peritumoral brain edema (PTBE). PTBE impacts the surgical management of intracranial meningiomas and is associated with a higher morbidity^2^ due to an increased risk of perioperative raised intracranial pressure, seizures,^3^ postoperative hematoma, and neurological deficits.^4,5^ In addition to apparent diffusion coefficient and tumor sphericity, PTBE represents one of the radiologic and radiomic characteristics that may predict meningioma grade and outcome.^6^ Numerous studies have attempted to identify specific risk factors for the development of PTBE (age, sex, histological grading and subtype, vascularity, blood supply, tumor size, expression of sex hormones, and vascular endothelial growth factor [VEGF] expression), but their results have been inconsistent.^2,7–13^ Hence, the pathogenesis of PTBE is still a matter of debate and relies on 4 main theories, which are probably associated at different levels: the compression theory (large meningiomas compressing the adjacent brain, leading to ischemia and cytotoxic edema), the secretory-excretory theory (specific histological secretory subtypes producing eosinophilic and periodic acid–Schiff positive inclusions), the venous compression theory (meningiomas obstructing venous outflow), and the hydrodynamic theory (tumor hypoxia leading to the secretion of angiogenic factors with leakage of plasma proteins and alterations of extracellular matrix).

Recent histopathological studies have highlighted the critical role of angiogenic factors (VEGF, hypoxia-inducible factor-1, platelet-derived growth factor, etc.) in the alterations of the peritumoral microcirculation network, emphasizing the responsibility of the hydrodynamic theory in the pathogenesis of PTBE in meningiomas. In parallel, perfusion and vascular macroscopic imaging studies resulted in questions about whether the impact of ischemic foci within the PTBE was secondary to the brain compression by a large meningioma or to the PTBE itself. The lack of adequate techniques to investigate the microcirculation in vivo has been a major limitation to explore and confirm these anomalies at the microscopic level. Only macroscopic imaging techniques (perfusion CT/MRI, xenon-enhanced CT, single-photon emission computed tomography, angiography, etc.) were used to investigate the peritumoral vascular network in meningiomas pointing some perfusion anomalies.^7,10,11,14–23^ Nevertheless, these macroscopic alterations of peritumoral circulation may not reflect the anomalies at the level of the microcirculation.^24,25^ The recent development of handheld noninvasive videomicroscopic techniques has offered new possibilities in the investigation of microcirculation.^26,27^

The aim of this study was to explore in vivo the peritumoral cerebral microcirculation and determine whether specific alterations of the microcirculation in PTBE are present in patients with intracranial meningiomas in terms of vessel density and blood flow. The effect of microsurgical resection on the peritumoral microcirculatory parameters and hence the responsibility of mass effect versus edema on peritumoral microcirculation was also investigated. To our knowledge, this is the first study showing firsthand evidence of alterations of the peritumoral cerebral microcirculation using in vivo sidestream dark-field (SDF) imaging.

## Material and Methods

### Patient Population

The intraoperative use of SDF imaging was approved by the Local Ethics Committee, and written informed consent was obtained from each patient. Our study population included 15 adult patients who were diagnosed with suspected intracranial convexity WHO grade I meningioma between January 2019 and September 2019. We included only meningiomas that developed on the skull convexity to allow the exposition of the adjacent cortical brain surface for microcirculation intravital imaging. To ensure patient homogeneity, recurrent meningiomas were excluded as well as WHO grade II and III meningiomas.

### Neuroimaging

**Figure 1.**
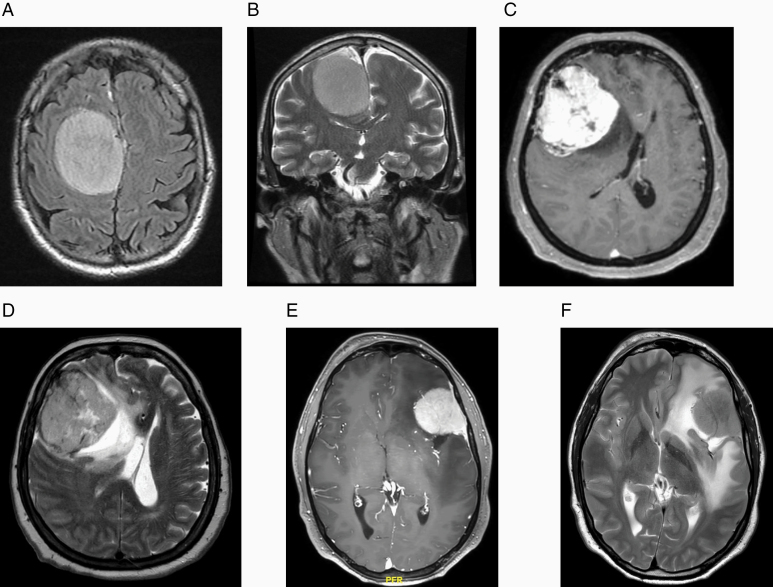
Peritumoral edema was classified according to Trittmacher criteria: grade 0 (A, axial Flair sequence and B, coronal T2-weighted sequence); grade 1 (C, axial T1-weighted sequence with gadolinium and D, axial T2-weighted sequence); grade 2 (E, axial T1-weighted sequence with gadolinium and F, axial T2-weighted sequence).

The tumor volume was evaluated on T1-weighted sequences with gadolinium using the formula for a spheroid (*V* = 4/3 πabc). PTBE was evaluated on T2-weighted sequence and was classified in terms of extension into 3 levels according to the Trittmacher criteria^28^ (Figure 1): grade 0 represents the absence of edema or the presence of a small halo around it; grade 1, moderate edema around the tumor; and grade 2, spread of edema involving the white matter of the affected hemisphere. Patients were split into 2 groups depending upon the presence of peritumoral edema (grades 1 and 2, E group) or the absence of peritumoral edema (grade 0, NE group).

### Operative Procedure

All patients underwent microsurgical gross total removal under neuronavigation by the same senior neurosurgeon (M.B.). Only 2 patients, who had grade 2 edema, received perioperative corticosteroids because of the presence of severe headaches. All patients neither underwent preoperative endovascular embolization nor received hyperosmolar solutions during the surgical procedure. Anesthetic operative parameters were maintained as stable as possible during the microcirculation imaging in all patients with objectives of normocapnia and a normal mean arterial pressure. All patients had preoperative hematocrit levels within a normal range, and none required transfusion during surgery. No static brain retraction was used in any patient.

### Microcirculation Registration

**Figure 2.**
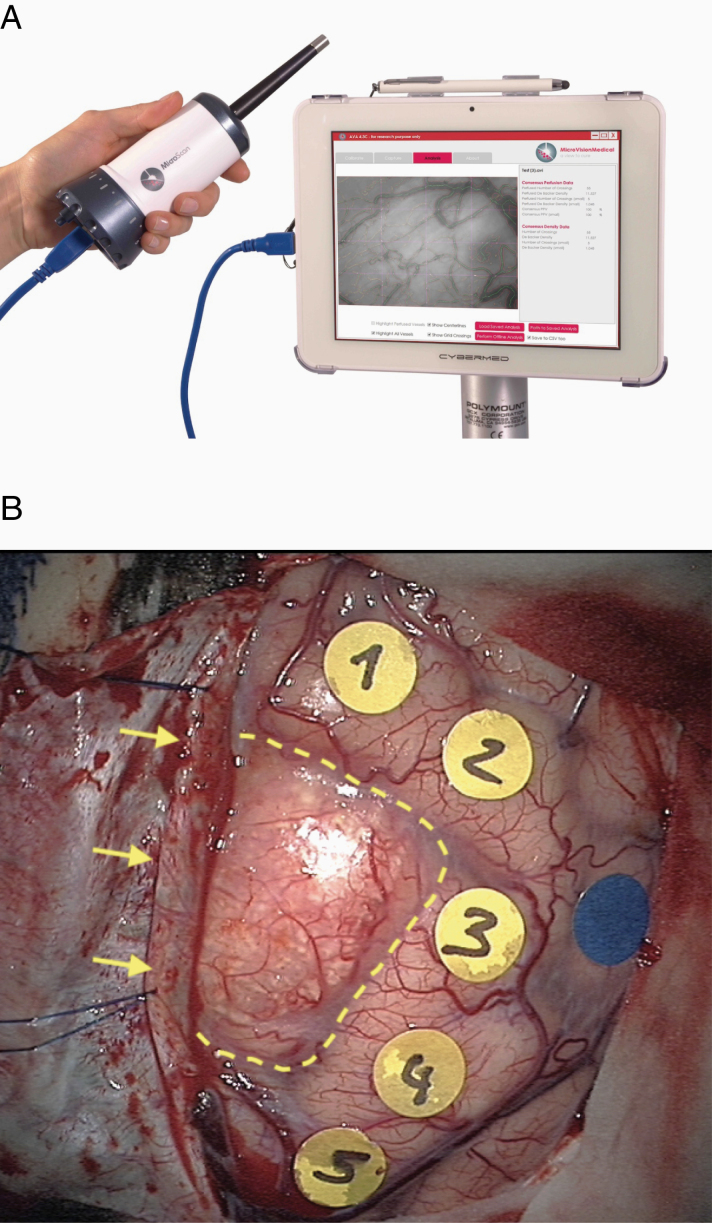
SDF imaging device used intraoperatively with a sterile draping (courtesy of Microscan; Microvision Medical) (A). Operative view of a parasagittal right frontal meningioma (dotted yellow line). The dura is opened and reflected on the sagittal sinus (yellow arrows). Five cortical spots (1–5) are identified within the adjacent peritumoral area corresponding to the peritumoral edema in accordance with the neuronavigation guidance. The blue spot is chosen as far from the tumor as the craniotomy allowed and served as a reference point for SDF imaging (B).

In all our patients, we assessed the peritumoral microcirculation parameters with SDF imaging (Microscan; Microvision Medical) before and after surgical resection. SDF imaging relies on a stroboscopic light-emitting diode ring-based modality integrated in a handheld device (Figure 2A).^24,29–32^ This device is composed of a light guide with a magnifying lens and an analogue embedded camera. The illumination comes from a green light with a 530 nm wavelength that is specifically absorbed by hemoglobin. Hence, the red blood cells appear as dark globules surrounding the vessels in a white/gray background. This technique provides visualization of the microcirculation at a depth of approximately 500 µm. We placed the tip of the device in a specifically designed sterile transparent plastic cap. The probe was stabilized in a self-retaining adjustable Leyla retractor arm to avoid hand-related micro-movements during registration and coupled with neuronavigation to select the peritumoral areas where the edema reaches the brain surface.

**Figure 3.**
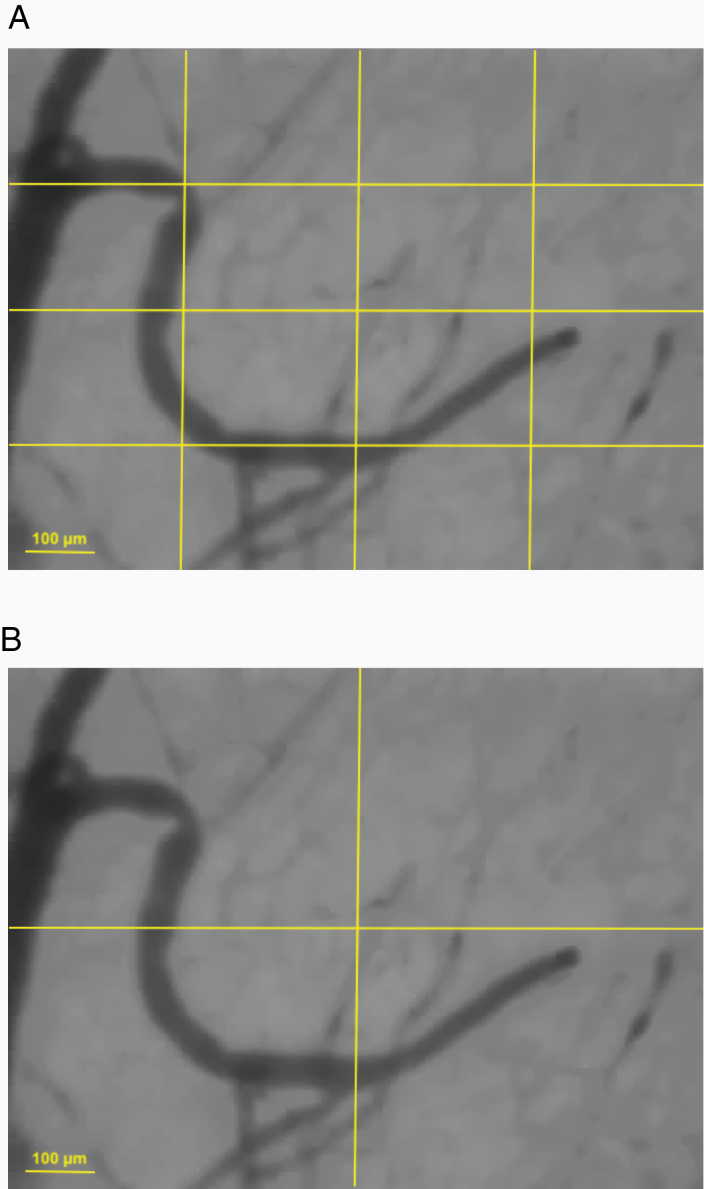
Determination of De Backer’s score (A) and microvascular flow index (B).

The craniotomy was performed in a standard fashion. Microcirculation evaluation was performed according to the consensus recommendations for the assessment of microcirculation.^33^ Six regions of interest (ROIs) were identified on the cortical surface around the meningioma and correlated to the neuronavigation data on T2 sequences: 5 ROIs immediately adjacent to the tumor and corresponding to the peritumoral region, and one control ROI was as far from the meningioma limit as the craniotomy allowed and where the edema is absent according to neuronavigation, to serve as “normal/control” microcirculation reference (Figure 2B). An SDF probe was placed at the contact of the cortex before any arachnoidal opening for pre-resection measurements (“baseline data”) and then at the end of the resection using the same ROIs (“post-resection data”). For each ROI, 3 video clips of 20 seconds were obtained. Particular attention was required to avoid any pressure artifact on the cortex and limit the quantity of fluid (either CSF or saline serum irrigation) between the cortical surface and the lens. During registration, the device was automatically adjusted as to have an optimal contrast and focus as well as a constant zoom for all registrations. The final on-screen magnification of the images was 325 times that of the original, corresponding to a field size of 1280 μm × 960 μm. AVA 3.0 software (AMC, University of Amsterdam, the Netherlands) was used to calculate the microcirculatory parameters:

- De Backer score (/mm): Number of vessels crossing 3 horizontal and 3 vertical equidistant lines (Figure 3A) divided by the total length of the lines, thus evaluating vessel density.
- Microvascular flow index (MFI): This index reflects the perfusion quality. The image is divided into 4 quadrants (Figure 3B) and the prominent type of flow is semi-quantitatively assessed by the observer using an ordinal scale in each quadrant (absent = 0, intermittent = 1, sluggish = 2, and normal = 3). MFI results from the average of the 4 quadrants. Absent flow corresponds to the complete lack of any flow throughout the video sequence. Intermittent flow is defined by at least half of the sequence without any flow. Sluggish is a continuous but very slow flow (Supplementary Video 1).
- Total vessel density (TVD/mm.mm^2^): The total length of the vessels divided by the total surface area of the analyzed image. A similar measurement was applied to small vessels with a diameter ≤20 µm, corresponding to the small vessel density (SVD).
- Perfused vessel density (PVD/mm.mm^2^): This value corresponds to the functional vessel density and is calculated from the total length of perfused vessels (MFI score ≥2) divided by the surface area of the analyzed image.
- Proportion of perfused vessels (PPV): Defined as the percentage of all visible vessels with at least a sluggish flow (100 × number of perfused vessels/total number of vessels).

### Statistical Analysis

Data on microcirculatory parameters are presented as the mean ± SD for each group of patients unless otherwise stated. Fisher’s exact test was used when appropriate. Differences between groups were compared using a paired Student’s *t* test if the data were normally distributed; otherwise, a Mann–Whitney *U* test was used. A Wilcoxon signed-rank test with continuity correction was used specifically for MFI and PPV. A *P* value of less than .05 was considered significant. Statistical analyses were conducted using R software version 3.5.1.

## Results

### Patient Characteristics

Three patients were excluded after pathological examination confirmed WHO grade II meningiomas. The 12 remaining patients with confirmed WHO grade I meningiomas were split into 2 groups of 6 patients each. The E group included patients with meningiomas with peritumoral edema (4 patients with Trittmacher grade 1 edema and 2 patients with grade 2 edema), and the NE group comprised meningiomas without peritumoral edema (Trittmacher grade 0). There were no significant differences between the characteristics of the 2 groups, except for the duration of symptoms (defined as the interval between the first symptoms and the imaging diagnosis), with 43.33 ± 13.54 days (mean ± SD) for the NE group and 14.16 ± 5.69 for the E group (*P* = .0033). The mean age was comparable in both groups, 55.16 ± 9.37 years old versus 50 ± 14.13 years old for the NE and E groups, respectively (*P* = .5133). The sex ratio (M:F) was 4:2 in the NE group and 3:3 in the E group. The tumor volume ranged from 8 to 45.98 cm^3^, and there was no significant difference between the groups (21.73 ± 7.4 cm^3^ for the NE group and 21.07 ± 12.61 cm^3^ for the E group, *P* = .922). Clinical presentation included significant unusual headaches in 8 patients (66.6%), mainly in the E group; motor deficits in 3 patients (25%: 2 patients with severe hemiparesis and 1 patient with left arm monoparesis); cognitive decline in 2 patients (16.6%); and one inaugural generalized seizure in 1 patient (8.3%) treated with oral Levetiracetam (500 mg twice daily). Neuroimaging data were representative of common grade I meningiomas: 50% of tumors were hypo-T2, 33% iso-T2, and 17% hyper-T2. Diffusion and perfusion data were without particularities (mean relative cerebral blood volume [rCBV] 7.4 ± 4.2 in the NE group vs 6.8 ± 2.4 in the E group, excluding one patient in whom perfusion data were not interpretable because of artifacts). A gross total microsurgical resection was possible in all patients. The brain–tumor interface was smooth in all patients in the NE group, while it was irregular and without an arachnoid plane in 33% of patients in the E group. The postoperative course was uneventful for all patients. The neurological outcome was excellent in all patients with resolution of increased intracranial pressure symptoms and complete recovery of any preoperative neurological deficits within 2 postoperative months. No patients had suffered postoperative seizures including the one who presented with an inaugural generalized seizure (Engel class I) in whom Levetiracetam has been progressively stopped at the sixth postoperative month. Pathological examination confirmed WHO grade I meningiomas in all patients without any particular distribution (50% meningothelial, 33.3% transitional, 8.3% fibrous, and 8.3% angiomatous).

### Microcirculatory Data at Baseline

Microcirculatory measurements at the reference point as far as possible from the meningioma were similar in both the NE and E groups at baseline (ie, before surgical resection). Thus, the mean values of reference points of the 2 groups were considered as representing “normal” cerebral cortical microcirculation and served as control values for subsequent comparisons.

In the NE group (Supplementary Table S1), we observed a statistically minor nonsignificant decrease in all microcirculatory parameters, particularly vessel density (De Backer score, TVD/SVD). Only MFI reached statistical significance (*P* = .048), whereas the other perfusion parameters did not (PVD and PPV).

In contrast, all microcirculatory parameters in the E group were significantly affected in the PTBE area with an approximately 50% drop compared to control (Supplementary Table S2). Vessel density was particularly reduced for small vessels (diameter <20 µm) with a decline of more than 68% of SVD values compared to control. Perfusion parameters were also very altered in PTBE with a drop of 46%, 76.5%, and 48.6% in the values of MFI, PVD, and PPV, respectively.

**Figures 4.**
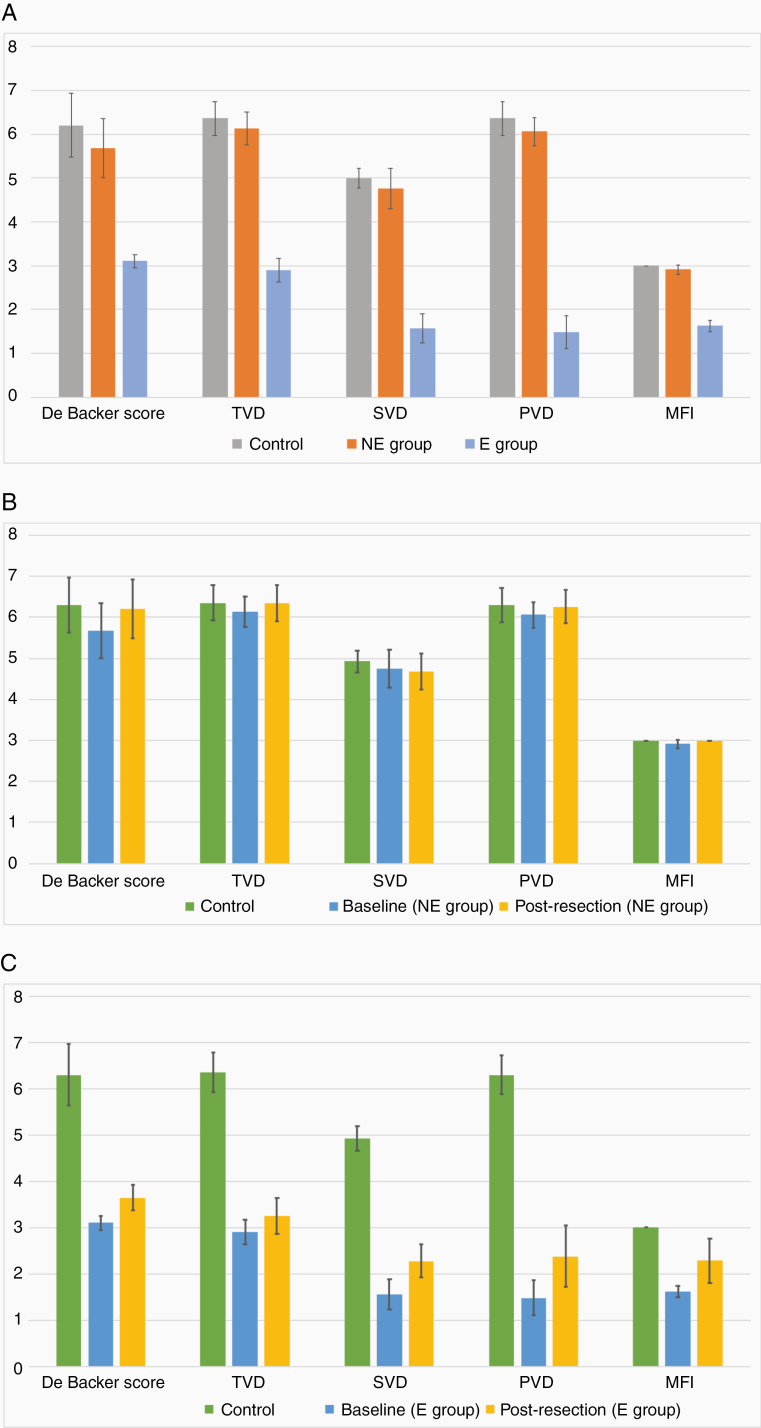
(A) Microcirculatory parameters in peritumoral area at baseline in NE and E groups. Control group corresponds to microcirculatory parameters at the reference point considered as normal cortical microcirculation. De Backer score is expressed in per millimeter. MFI: mean flow index (from 0 to 3), TVD: total vessel density (/mm.mm^2^), SVD: small vessel density (/mm.mm^2^), PVD: perfused vessel density (/mm.mm^2^). Mean ± SD. (B) Evolution of microcirculatory parameters of PTBE after surgical resection of meningiomas in the NE group, as compared to baseline (pre-resection parameters) and to the control group (normal cortical microcirculation). De Backer score is expressed in per millimeter. MFI: mean flow index (from 0 to 3), TVD: total vessel density (/mm.mm^2^), SVD: small vessel density (/mm.mm^2^), PVD: perfused vessel density (/mm.mm^2^). Mean ± SD. (C) Evolution of microcirculatory parameters of PTBE after surgical resection of meningiomas in E group, as compared to baseline (pre-resection parameters) and to the control group (normal cortical microcirculation). De Backer score is expressed in per millimeter. MFI: mean flow index (from 0 to 3), TVD: total vessel density (/mm.mm^2^), SVD: small vessel density (/mm.mm^2^), PVD: perfused vessel density (/mm.mm^2^). Mean ± SD.

Globally, microcirculatory patterns of the peritumoral area in the absence of edema (NE group) were almost similar to normal cortical microcirculation (apart from a slight decrease that may be due to mass effect), whereas all parameters (including perfusion and vessel density) were statistically significantly collapsed in PTBE (Figure 4A).

In no patient did we observe any pattern of angiogenic architecture in the limits of the 5 ROIs chosen for SDF imaging, namely, no irregular and tortuous vessels and no vascular conglomerates between normal microcirculation areas.^34^

### Microcirculatory Data After Tumoral Resection

In the NE group, surgical resection of the meningioma allowed for minor improvement of almost all microcirculatory parameters in the peritumoral area (Supplementary Table S3); however, they were not statistically significant, except for the De Backer score, which reached the value of the control reference measurement (6.21 ± 0.71/mm). This evolution may be interpreted as the direct effect of the elimination of the tumoral mass effect on the adjacent brain tissue (Figure 4B).

In the E group, the removal of the tumoral mass effect provoked a statistically significant increase of all microcirculation parameters (Supplementary Table S4), but the post-resection values remained considerably inferior to the normal reference pattern, reflecting only the consequence of the withdrawal of mass effect. The substantial residual difference between post-resection microcirculatory parameters and normal values (Figure 4C) may be the result of the peritumoral edema itself, confirming its prevalent role in the alterations of microcirculatory parameters in PTBE when compared to the mass effect alone, particularly when considering the SVD.

## Discussion

Until now, preoperative imaging techniques have revealed only macroscopic perfusion anomalies within the edema surrounding intracranial meningiomas, but there was no evidence of any vascular density or perfusion anomalies at the microcirculatory scale. Our study demonstrates for the first time in vivo substantial alterations of the microcirculatory parameters in the immediate environment of intracranial meningiomas, particularly when peritumoral edema exists. Hence, the significant decrease in both perfusion and vessel density in the PTBE as shown confirms the microvascular frailty of this area, and the necessity to preserve it from any excessive surgical manipulation and brain retraction to avoid ischemic damage.

### Imaging of Brain Microcirculation

Microcirculation is defined as a vast vascular network of small vessels with a diameter of less than 100 µm (capillaries, venules, and arterioles), ensuring not only the transport of vital substrates to tissues and clearing their waste products but also playing a critical role in vascular hemodynamics and resistance, blood coagulation, inflammation, and immunity.^27,34–41^ Direct visualization of the microcirculatory network has been described in 1987 by Slaaf et al.,^42^ and then it was improved in 1999 by Groner et al.^43^ with the development of orthogonal polarization spectral (OPS) imaging, the precursor to SDF imaging. The first human studies were focusing mainly on the alterations of microcirculation during sepsis by measurement on the sublingual mucosa.^25,39,40^ SDF was developed by Goedhart et al.^30^ in 2007 to overcome the drawbacks of OPS imaging, which was mainly the limited visualization of small capillaries due to blurring. Moreover, SDF imaging uses low-power LED, allowing for a better autonomy and portability for clinical and operative use. SDF imaging offers a precise estimation of the functional capillary density.

The development of handheld compact devices for OPS imaging and then SDF imaging has opened a large field of possibilities, including for the investigation of cerebral microcirculation.^43^ In 2000, Uhl et al.^34^ confirmed the feasibility of OPS imaging in a series of 12 patients (4 incidental aneurysms serving as control group, 3 patients with aneurysmal subarachnoid hemorrhage [SAH], and 5 patients with brain tumors). They were able to visualize vasospasm in small arterioles (diameter ≤150 µm) in patients with SAH undergoing normal transcranial Doppler imaging and in the absence of clinical signs of vasospasm. Pennings et al.^44^ showed with OPS imaging the increased arteriolar contractility in response to hyperventilation in 16 patients who underwent an operation for aneurysmal SAH. After the introduction of SDF imaging in 2007,^30^ very few human brain microcirculation studies were published (Table 1). In 2011, Pérez-Bárcena et al.^29^ applied SDF imaging to the evaluation of cortical microcirculation in malignant stroke patients requiring decompressive craniectomy. They were able to confirm a significant blood flow reduction in the cortical microcirculation and decreased vascular density in patients with stroke. To our knowledge, our study demonstrates for the first time in vivo specific microcirculation alterations within the PTBE. Until now, preoperative imaging techniques have revealed only perfusion anomalies within the edema surrounding intracranial meningiomas, but there was no evidence of any vascular density anomaly as well. We were able to confirm at the microscopic level the alterations of vascular density in the PTBE likewise perfusion anomalies. The very significant decrease in both the perfusion and vessel density in the PTBE as shown confirms the vulnerability of this zone toward any surgical manipulation, particularly the use of a self-retaining retractor on the peritumoral brain tissue during the resection. This microcirculatory vulnerability of the PTBE as quantified in our study also raises the question of the necessity of exploring the influence of the systemic hemodynamic parameters during surgery, as well as the impact of vasoactive factors (mean arterial pressure, ETCO_2_, intravenous noradrenaline, etc.) on peritumoral microcirculation.

**Table 1.** Main Applications of OPS and SDF Techniques for Imaging the Brain Microcirculation

Authors (year)	Technique	Context	Patient Population	Main Conclusions
Uhl et al. (2000)	OPS	Study of the feasibility of OPS imaging during different neurosurgical procedures	12 patients (4 incidental aneurysms serving as control group, 3 patients with aneurysmal subarachnoid hemorrhage, and 5 patients with brain tumors)	• Visualization of vasospasm is small arterioles (diameter ≤150 µm) in patients with SAH having normal transcranial doppler and without a clinical sign of vasospasm • Evidence of an increase in functional capillary density at the end of the surgery probably because of a decrease in intracranial pressure either by CSF release or tumor debulking
Uhl et al. (2003)	OPS	Aneurysmal surgery (without and with SAH)	10 patients with aneurysmal SAH compared to a control group of 3 patients with incidental aneurysms	• Significant decrease of capillary density in patients with SAH (30.5 ± 13.8/cm in contrast with 91.5 ± 36.5/cm for patients with incidental aneurysms) • Microvasopasms on the small cortical arterioles with a reduction of diameter up to 75.1%
Pennings et al. (2004)	OPS	Aneurysmal SAH surgery	16 patients in 2 groups: early surgery group (within 48 h from bleeding) and late surgery group	• In the early surgery group provoked hypocapnia resulted in a decrease of 39 ± 15% in arteriolar diameter, while in the late surgery group the decrease was estimated to 17 ± 20% compared to 7 ± 7% in controls • Microvascular tonus is significantly increased after SAH, suggesting a potential role in the pathogenesis of vasospasm-induced ischemia
Pennings et al. (2006)	OPS	Imaging of the peri-nidal region in brain Arterio-Venous Malformation	2 patients with brain AVM—OPS imaging before and after surgical resection	• Significant increase in microvascular flow in the peri-nidal brain with an MFI raising from 2 to 3.7 and FCD raising from 1.4 ± 1.3 cm/mm^2^ to 2.1 ± 0.8 cm/mm^2^ • Data were consistent with the hyperperfusion syndrome and normal perfusion pressure breakthrough
Pennings et al. (2009)	OPS	Aneurysmal SAH surgery—response of brain microcirculation to topical papaverine	14 patients operated on with aneurysmal SAH versus 3 patients with deep pathology not affecting the cortical microcirculation (control group)	• Unpredictable response to topical papaverine with both dilatation and constriction observed • Risk of a rebound effect • Diminished vasodilatory capacity of the cerebral microcirculation after subarachnoid hemorrhage still to be confirmed
Pérez-Bárcena et al. (2011)	SDF	Cortical microcirculation in stroke	6 patients with stroke operated on for decompressive craniectomy versus 5 neurosurgical patients without cortical microcirculation pathology (control group)	Significant blood flow reduction in the cortical microcirculation and decreased vascular density in patients with stroke
Pérez-Bárcena et al. (2015)	SDF	Cortical microcirculation in traumatic brain injury (TBI)	14 patients with TBI requiring surgery (5 subdural hematomas and 9 parenchymal lesions) versus 5 neurosurgical patients without cortical microcirculation pathology (control group)	• PPV similar in all groups • Perfused vessel density index smaller in the peri-contusional area • Overall preservation of microcirculation parameters in TBI
Berhouma et al. (2020)	SDF	Microcirculation in the peritumoral brain edema in intracranial meningiomas	12 patients with intracranial WHO grade I meningiomas split into 2 groups according to the presence (E group) or absence (NE group) of peritumoral edema	• Severe alterations of vessel density and blood flow parameters in peritumoral edema • Partial recovery of microcirculatory parameters after surgical resection due to withdrawal of mass effect only • Hypothesis that the severity of microcirculatory alterations may predict the presence of ischemic spots and hence the neurological outcome

### Characterization of Peritumoral Perfusion

While peritumoral perfusion can be evaluated intraoperatively with several optical technologies (laser speckle contrast imaging, laser doppler imaging, intraoperative MRI, thermography, etc.), only SDF imaging provides a direct morphological assessment of vessel density and the proportion of perfused vessel in addition to a semiquantitative estimation of blood flow. Until now, the peritumoral perfusion in meningiomas has been studied preoperatively using macroscopic imaging mostly MRI. MR perfusion sequences within the peritumoral brain area remain difficult to interpret mainly because of the poor resolution and the mass effect. Thus, in our series, preoperative MR perfusion was measured only within the meningioma as we were not able to obtain interpretable macroscopic perfusion data within the peritumoral area. Very rare studies have evaluated MR perfusion within the PTBE.^45^ It has been demonstrated that the rCBV is significantly increased in PTBE in WHO grade II and III meningiomas compared with grade I, which is probably because of tumor pial invasion and local brain angiogenesis.^19^ Based on these observations, we chose to study only WHO grade I meningiomas to avoid major imaging interference from peritumoral neo-vascularization.

In WHO grade I meningiomas, variations of rCBV in PTBE were the result of different factors, mainly vasogenic edema, angiogenesis, and ischemia secondary to tumoral mass effect. It is still unclear whether one of these factors prevails or is the consequence of the other factors in the pathogenesis of PTBE, even if ischemia appears as a secondary event. Ischemic alterations in the peritumoral brain in meningiomas have been investigated in detail and so has the role of mass effect.^17,45^ Lehmann et al.^18^ studied the PTBE in meningiomas with MR perfusion imaging and confirmed an increase in relative maximum signal drop with rising distance from the meningioma, suggesting the role of compression by the mass effect, as demonstrated in our study through the evolution of microcirculatory parameters before and after surgical resection. Tatagiba et al.^23^ provided a study of 12 intracranial meningiomas using a xenon-enhanced CT scan. They found CBF values 28% lower in the peritumoral area compared to the ipsilateral hemisphere. According to the authors, the low peritumoral blood flow in meningiomas is not only secondary to vasogenic edema but also results from an ischemic phenomenon due to the mass effect, thus explaining the absence of response to corticosteroids and occasionally definitive postoperative deficits. In our E group, the significant residual microcirculatory alterations after surgical resection were probably secondary to the combined effect of edema and ischemic modifications. We can hypothesize that ischemic changes in the PTBE may result from both vessel density drop as well as perfusion anomalies translating into low MFI and PPV values. The proportion between irreversible ischemic changes and reversible edema in PTBE may condition the postoperative neurological outcome. In our future SDF imaging work, we aim to study the correlation between the degree of in vivo microcirculatory alteration severity within the PTBE in edematous meningiomas and the postoperative neurological outcome to verify if the in vivo peritumoral microcirculatory pattern can eventually predict the risk of definitive neurological deficits. Furthermore, we may be able to determine if ischemic irreversible alterations are more specifically correlated with either vessel density or blood flow anomalies. These ischemic anomalies in PTBE were also confirmed at a macroscopic level in an MR study by Domingo et al.^46^ The authors showed low CBV with elevated lactate in the PTBE, suggesting that local hypoperfusion and metabolic disturbances may play a potential role in the pathogenesis of PTBE. Bitzer et al.^45^ explored 26 patients with 27 meningiomas and 5 gliomas with perfusion-weighted imaging. The authors concluded that there was no decrease; rather, there was a slight increase in perfusion in peritumoral brain tissue in meningiomas without edema. Though in meningiomas with edema, these perfusion parameters were significantly reduced. Therefore, Bitzer et al. raised the question of whether peritumoral edema caused decreased perfusion or if the reduction in perfusion generated edema. In their study, there were no anomalies in diffusion and perfusion in the peritumoral brain in meningiomas without edema, whereas they demonstrated areas of ischemia with a reduction of Apparent Diffusion Coeffcient values in about one-third of meningiomas with edema. The authors noted that these ischemic foci were very limited in size compared to the extent of edema, and they always occurred immediately adjacent to the tumor wall. Since two-thirds of the meningiomas with edema did not exhibit these ischemic features, the authors concluded that ischemia should be regarded as a secondary event in the pathogenesis of PTBE.

### Limitations and Perspectives

We aimed to assess the feasibility of intraoperative SDF imaging in meningiomas and to confirm the existence of both perfusion and vessel density alterations in PTBE. A larger series correlating in vivo SDF imaging and preoperative high-definition perfusion MR imaging should confirm our data and moreover explore the ischemic responsibility in the pathogenesis of PTBE. The influence of intraoperative systemic factors (mean arterial pressure, vasoactive drugs, and ETCO_2_) on microcirculation in PTBE during surgery may also be evaluated using SDF imaging in larger series.

Notwithstanding we used neuronavigation to perform measurements at the peritumoral locations where the edema was the most superficial, we remained limited to the assessment of the peritumoral cortex, whereas deep peritumoral regions were not accessible. Therefore, our results may not reflect the whole microcirculatory environment of the tumor, which may be heterogeneous, particularly if the majority of peritumoral edema is located deep in the brain tissue not reachable by the SDF registration capacity.

**Figure 5.**
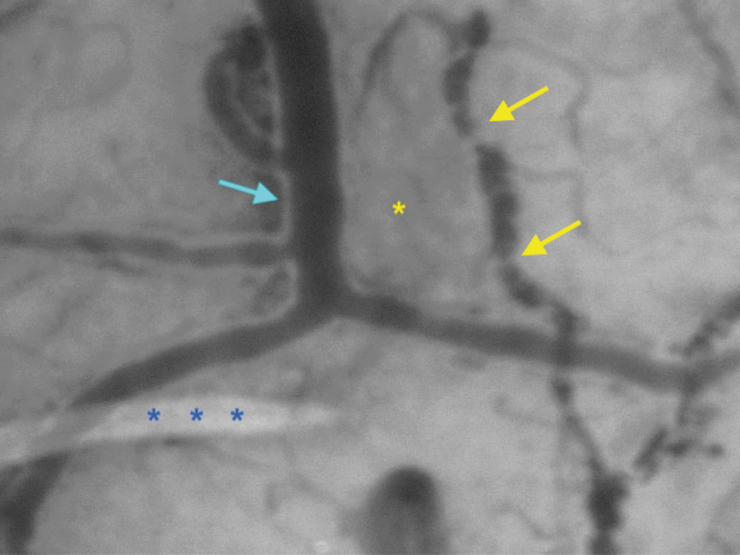
Example of SDF imaging (Patient N°4) depicting the importance of the quality of image acquisition to ensure reliable microcirculation parameters and avoid artifacts: A slight local subarachnoid hemorrhage (yellow asterisk) secondary to the craniotomy may alter the image contrast. In this case, wall vessel normally not visible in SDF imaging becomes visible (blue arrow). The presence of fluids between the lens and the cortical surface should also be avoided and may interfere with the visibility of small arterioles distorting the vessel density index (blue asterisks). Please note the sluggish flow pattern (MFI:2) resulting in very low columns of globular erythrocytes (yellow arrows).

SDF imaging is a contact method requiring an optimal interface with the brain’s surface. Pressure-induced microcirculatory alterations are possible and may impair the accuracy of the measured parameters (Figure 5). Noncontact methods are available (laser speckle contrast imaging, thermography, etc.), allowing for the analysis of larger areas but remain sensitive to motion artifacts and do not provide morphological information at the microcirculatory scale (TVD, PPV, etc.).

Other technological refinements that may facilitate intraoperative microcirculation imaging include shorter data acquisition with automatized analysis and an incident light beam probe (incident dark-field imaging) that could potentially offer a better image resolution.^47^

## Conclusions

Even though our study was limited to very few patients, it has clearly confirmed the feasibility of in vivo SDF microcirculation imaging in brain surgery and showed quantitatively severe alterations of both perfusion and vessel density parameters in PTBE, confirming the vulnerability of the peritumoral microcirculation in edematous meningiomas. Whether the severity of these alterations may correlate with ischemic foci in PTBE and thus predict the postoperative neurological outcome is still not known. Obviously, SDF imaging opens new insights in the exploration of brain microcirculation.

## Supplementary Material

vdaa108_suppl_Supplementary_Table_S1

vdaa108_suppl_Supplementary_Table_S2

vdaa108_suppl_Supplementary_Table_S3

vdaa108_suppl_Supplementary_Table_S4

vdaa108_suppl_Supplementary_Video

vdaa108_suppl_Supplementary_Video_Caption

## Notes

**Authorship Statement.** All authors have critically revised the manuscript and approved its final content. Manuscript draft, analysis, and interpretation of the data: M.B. and A.C.L. Acquisition of the data: M.B., T.P., C.D., and J.G. Conception and design of the study: M.B., J.G., I.P.G., O.E., D.M., J.H., F.D., and F.C. Interpretation of the data: M.B., A.C.L., and F.C.
