# Supplementary material for: Alterations of cerebral microcirculation in peritumoral edema: feasibility of in vivo sidestream dark-field imaging in intracranial meningiomas
Source: Neurooncol Adv. 2020 Aug 27;2(1):vdaa108. doi: 10.1093/noajnl/vdaa108 (PMC7542984; doi:10.1093/noajnl/vdaa108)
Supplement: vdaa108_suppl_Supplementary_Table_S1 [file vdaa108_suppl_supplementary_table_s1.docx]

|  | Reference point (control) | Peri-tumoral area  (n=6) | p |
| --- | --- | --- | --- |
| De Backer score (mm^-1^) | 6.30 ± 0.66 | 5.68 ± 0.67 | 0.1173 |
| MFI | 3 | 2.91 ± 0.11 | **0.0481** |
| TVD (mm.mm^-2^) | 6.35 ± 0.43 | 6.13 ± 0.37 | 0.3271 |
| SVD (mm.mm^-2^) | 4.93 ± 0.27 | 4.75 ± 0.46 | 0.4408 |
| PVD (mm.mm^-2^) | 6.30 ± 0.42 | 6.06 ± 0.32 | 0.2353 |
| PPV (%) | 99.35 ± 1.17 | 98.96 ± 1.46 | 0.6782 |

Table S1. Microcirculatory parameters at the reference point (control) at baseline compared with the peri-tumoral area in the NE group.

MFI: Mean flow index, TVD: Total vessel density, SVD: Small vessel density, PVD: Perfused vessel density, PPV: Proportion of perfused vessels
