# Supplementary material for: Alterations of cerebral microcirculation in peritumoral edema: feasibility of in vivo sidestream dark-field imaging in intracranial meningiomas
Source: Neurooncol Adv. 2020 Aug 27;2(1):vdaa108. doi: 10.1093/noajnl/vdaa108 (PMC7542984; doi:10.1093/noajnl/vdaa108)
Supplement: vdaa108_suppl_Supplementary_Table_S2 [file vdaa108_suppl_supplementary_table_s2.docx]

|  | Reference point (control) | PTBE  (n=6) | p |
| --- | --- | --- | --- |
| De Backer score (mm^-1^) | 6.30 ± 0.66 | 3.1 ± 0.15 | **< 0.0001** |
| MFI | 3 | 1.62 ± 0.12 | **< 0.0001** |
| TVD (mm.mm^-2^) | 6.35 ± 0.43 | 2.9 ± 0.27 | **< 0.0001** |
| SVD (mm.mm^-2^) | 4.93 ± 0.27 | 1.56 ± 0.33 | **< 0.0001** |
| PVD (mm.mm^-2^) | 6.30 ± 0.42 | 1.48 ± 0.38 | **< 0.0001** |
| PPV (%) | 99.35 ± 1.17 | 51.06 ± 11.62 | **0.0003** |

Table S2. Microcirculatory parameters at the reference point (control) at baseline compared with the peri-tumoral brain edema (PTBE) area in the E group.

MFI: Mean flow index, TVD: Total vessel density, SVD: Small vessel density, PVD: Perfused vessel density, PPV: Proportion of perfused vessels
