# Supplementary material for: Alterations of cerebral microcirculation in peritumoral edema: feasibility of in vivo sidestream dark-field imaging in intracranial meningiomas
Source: Neurooncol Adv. 2020 Aug 27;2(1):vdaa108. doi: 10.1093/noajnl/vdaa108 (PMC7542984; doi:10.1093/noajnl/vdaa108)
Supplement: vdaa108_suppl_Supplementary_Table_S3 [file vdaa108_suppl_supplementary_table_s3.docx]

|  | Baseline  (n=6) | Post-resection  (n=6) | p |
| --- | --- | --- | --- |
| De Backer score (mm^-1^) | 5.68 ± 0.67 | 6.21 ± 0.71 | **0.0044** |
| MFI | 2.91 ± 0.11 | 3 | 0.3457 |
| TVD (mm.mm^-2^) | 6.13 ± 0.37 | 6.35 ± 0.44 | 0.1152 |
| SVD (mm.mm^-2^) | 4.75 ± 0.46 | 4.68 ± 0.45 | 0.4441 |
| PVD (mm.mm^-2^) | 6.06 ± 0.32 | 6.26 ± 0.40 | 0.0667 |
| PPV (%) | 98.96 ± 1.46 | 98.72 ± 1.03 | 1 |

Table S3. Evolution of microcirculatory parameters in the peri-tumoral area after surgical resection in NE group.

MFI: Mean flow index, TVD: Total vessel density, SVD: Small vessel density, PVD: Perfused vessel density, PPV: Proportion of perfused vessels
