# Supplementary material for: Alterations of cerebral microcirculation in peritumoral edema: feasibility of in vivo sidestream dark-field imaging in intracranial meningiomas
Source: Neurooncol Adv. 2020 Aug 27;2(1):vdaa108. doi: 10.1093/noajnl/vdaa108 (PMC7542984; doi:10.1093/noajnl/vdaa108)
Supplement: vdaa108_suppl_Supplementary_Table_S4 [file vdaa108_suppl_supplementary_table_s4.docx]

|  | Baseline  (n=6) | Post-resection  (n=6) | p |
| --- | --- | --- | --- |
| De Backer score (mm^-1^) | 3.1 ± 0.15 | 3.65 ± 0.28 | **0.0066** |
| MFI | 1.62 ± 0.12 | 2.29 ± 0.48 | 0.0590 |
| TVD (mm.mm^-2^) | 2.9 ± 0.27 | 3.25 ± 0.39 | **0.0235** |
| SVD (mm.mm^-2^) | 1.56 ± 0.33 | 2.28 ± 0.35 | **0.0022** |
| PVD (mm.mm^-2^) | 1.48 ± 0.38 | 2.38 ± 0.67 | **0.0066** |
| PPV (%) | 51.06 ± 11.62 | 72.95 ± 17.09 | **0.0312** |

Table S4. Evolution of microcirculatory parameters in the PTBE after surgical resection in E group.

MFI: Mean flow index, TVD: Total vessel density, SVD: Small vessel density, PVD: Perfused vessel density, PPV: Proportion of perfused vessels
