# Supplementary material for: Alterations of cerebral microcirculation in peritumoral edema: feasibility of in vivo sidestream dark-field imaging in intracranial meningiomas
Source: Neurooncol Adv. 2020 Aug 27;2(1):vdaa108. doi: 10.1093/noajnl/vdaa108 (PMC7542984; doi:10.1093/noajnl/vdaa108)
Supplement: vdaa108_suppl_Supplementary_Video_Caption [file vdaa108_suppl_supplementary_video_caption.docx]

SUPPLEMENTARY MATERIAL

Supplementary Video 1

Microcirculation imaging in the TPBE (E group – Patient n°7) depicting the sluggish flow and decreased vascular density.
